# The roles and impacts of human hunter-gatherers in North Pacific marine food webs

**DOI:** 10.1038/srep21179

**Published:** 2016-02-17

**Authors:** Jennifer A. Dunne, Herbert Maschner, Matthew W. Betts, Nancy Huntly, Roly Russell, Richard J. Williams, Spencer A. Wood

**Affiliations:** 1 Santa Fe Institute, 1399 Hyde Park Road, Santa Fe, NM 87501, USA; 2 Center for Virtualization and Applied Spatial Technologies, University of South Florida, 4202 E. Fowler Ave., NES 107, Tampa, FL 33620; 3 Canadian Museum of History, 100 Laurier Street, Gatineau, QC K1A 0M8, Canada; 4 Ecology Center, Utah State University, 5205 Old Main Hill, Logan, UT 84322-5205, USA; 5 The Sandhill Institute for Complexity and Sustainability, Grand Forks, British Columbia, Canada; 6 VibrantData Inc., 943 Clay Street, San Francisco, CA 94108; 7 The Natural Capital Project, Stanford University, 371 Serra Mall, Stanford, CA 94305, USA; 8 School for Environmental and Forest Sciences, 4000 15th Ave NE, University of Washington, Seattle, WA 98195, USA

## Abstract

There is a nearly 10,000-year history of human presence in the western Gulf of Alaska, but little understanding of how human foragers integrated into and impacted ecosystems through their roles as hunter-gatherers. We present two highly resolved intertidal and nearshore food webs for the Sanak Archipelago in the eastern Aleutian Islands and use them to compare trophic roles of prehistoric humans to other species. We find that the native Aleut people played distinctive roles as super-generalist and highly-omnivorous consumers closely connected to other species. Although the human population was positioned to have strong effects, arrival and presence of Aleut people in the Sanak Archipelago does not appear associated with long-term extinctions. We simulated food web dynamics to explore to what degree introducing a species with trophic roles like those of an Aleut forager, and allowing for variable strong feeding to reflect use of hunting technology, is likely to trigger extinctions. Potential extinctions decreased when an invading omnivorous super-generalist consumer focused strong feeding on decreasing fractions of its possible resources. This study presents the first assessment of the structural roles of humans as consumers within complex ecological networks, and potential impacts of those roles and feeding behavior on associated extinctions.

Most studies of the relationships of humans and ecosystems are presented in terms of human impacts *on* ecosystems^1^,^2^. However, our ability to understand and mitigate human impacts depends on research that elucidates the roles humans play *in* ecosystems including how they interact with other species^3^. In modern marine ecosystems, humans are depleting many commercial fisheries, causing major disruptions to ecosystem function and the persistence of species^1^,^4^,^5^,^6^,^7^. As a result, regulators have curtailed fisheries and excluded local peoples from traditional harvesting territories^2^. This is a critical problem for the Aleut peoples of the western Gulf of Alaska, who depend strongly on biotic resources from the marine communities^8^,^9^,^10^. This central dependence on marine resources stretches back throughout the nearly 10,000-year prehistory of human presence in the North Pacific, raising the questions of what kinds of impacts humans have had on marine species and ecosystems in this area and how should future impacts be managed. In general, the specific roles that local, prehistoric peoples have played in the structure and functioning of marine ecosystems are poorly documented and largely unknown or necessarily somewhat speculative.

Unlike in many terrestrial systems, where there is increasing evidence that prehistoric humans likely contributed directly and indirectly to the extinction of a number of species, there is little evidence for such extinctions in marine systems^11^. This does not mean that prehistoric humans had no significant impacts on marine ecosystems. For example, in the Aleutian Islands, there is evidence that through hunting of sea otters, prehistoric humans may have caused certain areas to switch from algal-dominated kelp-forest habitats into sea-urchin-dominated barrens devoid of macroalgae^12^, a well-documented trophic cascade. This in turn could have limited the habitat and population of Steller’s sea cows, which also were probably hunted or scavenged occasionally by prehistoric humans^13^. Steller’s sea cows ultimately went extinct by 1768 as a result of Russian arrival to the Aleutian Islands and the introduction of commercial hunting and fishing^14^.

The Sanak Island Biocomplexity Project was developed to investigate the integration of human foragers into marine ecosystems in the Western Gulf of Alaska^3^, a region considered one of the world’s last great fisheries. This region is inhabited by Aleut, descendants of the first people to inhabit the Aleutian Islands, which they colonized shorty after deglaciation. The greater far North Pacific ecosystem has experienced human subsistence harvesting for at least 10,000 years^15^, and except for a few isolated islands, the entire region was fully occupied by 7,000 years ago^16^. As a result, none of the modern fisheries in the region have a well-documented pre-fishing or pre-human baseline^7^,^11^, complicating management decisions. Sanak Island, with an approximately 7,000-year record of human habitation, is the largest island in a relatively isolated archipelago 50 km south of the mainland Alaska Peninsula, at the center of the Alaska Current, surrounded by some of the most important fisheries and sea mammal habitats in the entire Gulf of Alaska, and subject to a suite of competing management regimes. From the Russians removing the Aleut from the island to protect *Enhydra lutris* (sea otters) in 1824 to current fisheries bans to protect *Eumetopias jubatus* (Steller sea lions), it has also been the center of many endangered species conflicts. The Aleut moved from the Sanak Archipelago in the 1970s, but the nearshore waters are still harvested by indigenous commercial and subsistence foragers.

In this study, we integrated anthropological data and food web data, analyses, and modeling to explore aspects of socioecological structure and associated dynamics that may inhibit or facilitate ecosystem stability in this region. To identify and quantify the trophic roles of human foragers in western Gulf of Alaska marine ecosystems in terms of their topological positions within complex networks of feeding interactions among taxa, we compiled detailed, highly resolved food web data for the Sanak intertidal and nearshore ecosystems. These comprehensive, cumulative food webs represent the architecture of the feeding relationships among co-occurring taxa that lived or frequently fed in those marine habitats since human introduction. We then used simulations of trophic dynamics to explore to what degree the introduction of a species with human-like food web roles as well as a capacity for strong feeding is likely to disrupt ecological integrity via subsequent extinctions.

## Results and Discussion

### Human Roles in Sanak Marine Food Webs

**Figure 1:**
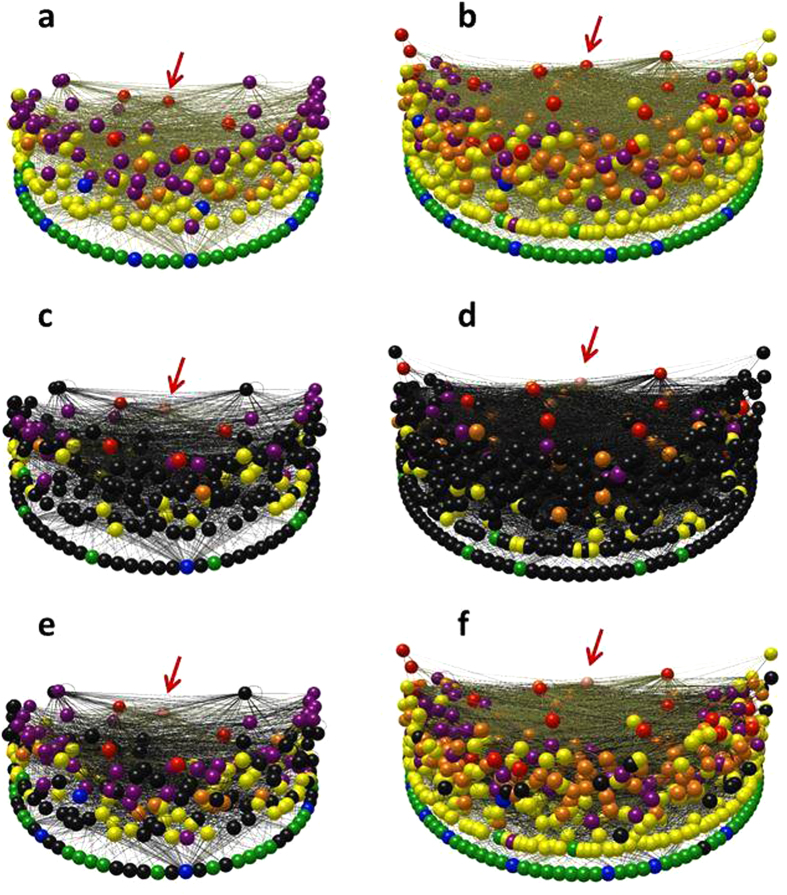
Sanak Archipelago food webs. Spheres indicate taxa, lines indicate feeding interactions. Vertical axis indicates trophic level, calculated using the “short-weighted trophic level” algorithm^76^. The maximum trophic level of a taxon in the intertidal and nearshore food webs is 3.98 and 4.94, respectively, and the webs’ mean trophic levels (Supplementary Table S1) are 2.44 and 2.83, respectively. Sphere color indicates type of taxon: green = algae, blue = miscellaneous (e.g., detritus, protozoa, bacteria, biofilm, lichen, seagrass), yellow = invertebrates, orange = fishes, red = mammals, purple = birds. Red arrows point to *Homo sapiens.* (**a)** Sanak intertidal web. (**b)** Sanak nearshore web. (**c,d)** Sanak intertidal and nearshore webs showing resources of *Homo sapiens* in color. (**e,f)** Sanak intertidal and nearshore webs showing taxa within two links of *Homo sapiens* in color. Images created with Network3D^72^,^73^, available freely by request to jdunne@santafe.edu.

**Figure 2:**
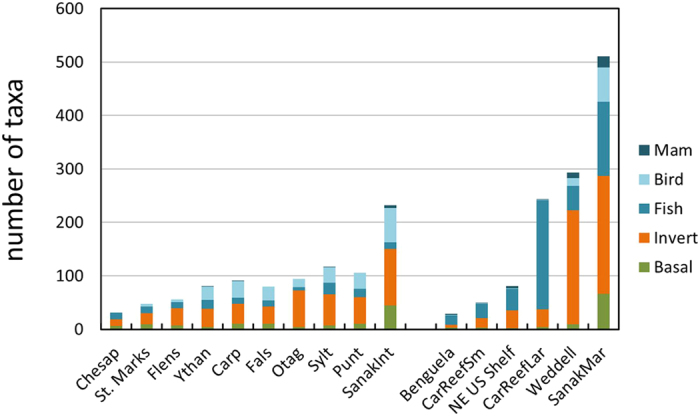
Comparison of diversity and types of taxa in marine food webs. First group shows data for ten coastal webs in order of increasing species richness: Chesap = Chesapeake Bay^62^; St. Marks = St. Marks Estuary^63^; Flens = Flensburg Fjord^64^; Ythan = Ythan Estuary^65^; Carp = Carpinteria Salt Marsh^66^; Fals = Bahia Falsa^66^; Otag = Otago Harbor^67^; Sylt = Sylt Tidal Basin^68^; Punt = Estero de Punta Banda^66^; SanakInt = Sanak Intertidal. Second group shows six marine webs in order of increasing species richness: Benguela = Benguela Fishery^69^; CarReefSm = Caribbean Reef, small version^70^; NE US Shelf = Northeast U.S. Shelf^17^; CarReefLar = Caribbean Reef, large version^70^; Weddell = Weddell Sea^71^; SanakMar = Sanak Nearshore. Vertebrate data shown in shades of blue, invertebrate in orange, basal in green. Data shown for trophic species versions of webs, where taxa with the same set of consumers and resources are aggregated into single nodes^60^. The same figure for original species versions of webs is given in Supplementary Figure S1.

The Sanak intertidal food web has 235 taxa and 1804 trophic links, and the Sanak nearshore food web has 513 taxa and 6774 links (Fig. 1a,b, Supplementary Table S1, Supplementary Data S1). They are among the largest coastal and marine food web datasets yet compiled and have unusually detailed resolution of invertebrates and basal (e.g., primary producers) taxa (Fig. 2, Supplementary Fig. S1, Supplementary Table S1). The Sanak webs are the most diverse and highly and evenly resolved food webs to include *Homo sapiens*. Most previously documented food webs with humans have fewer than 35 taxa and 60 links (Supplementary Table S2). The largest previous web we know of with humans included as a node is the Northeast US Shelf marine food web with 81 taxa and 1562 links^17^.

**Figure 3:**
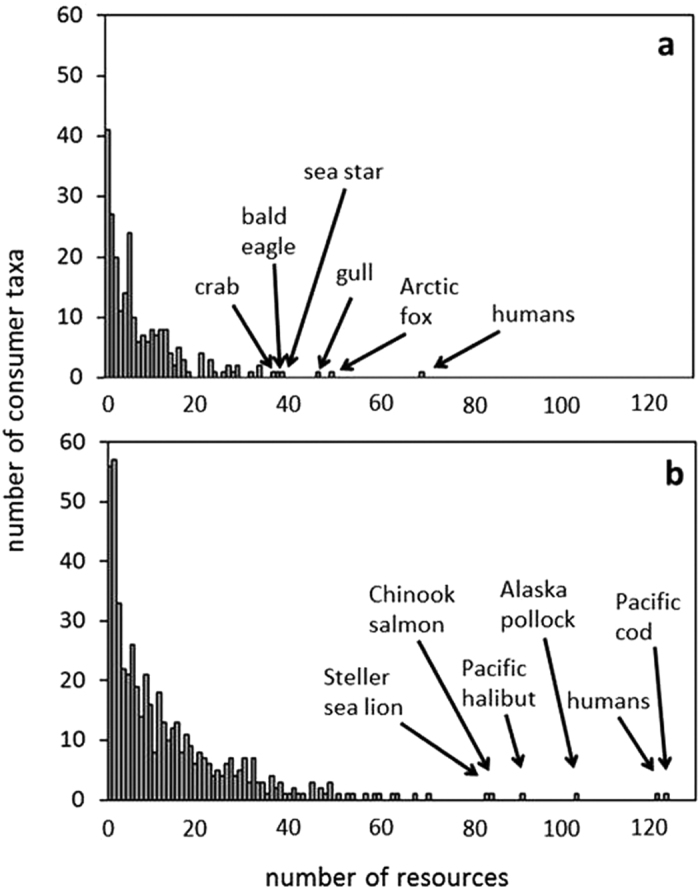
Histogram of number of resource taxa per consumer. The vertical axis shows the number of consumers in that web that have a particular number of resource taxa indicated along the horizontal axis. The six most generalist taxa are labeled with their common name. (**a)** Sanak intertidal web. (**b)** Sanak nearshore web.

The high quality of the Sanak food web data allows definitive, quantitative comparison of the structural trophic roles of *H. sapiens* to those of other species. Humans fed on 70 taxa in the Sanak intertidal web (29.8%), far exceeding the next most general consumer, *Vulpes* (arctic fox) with 50 resources. In the nearshore web, *Homo sapiens* fed on 122 taxa (23.8%), similar to *Gadus macrocephalus* (Pacific cod), which fed on 124 taxa, and far exceeding the next most general consumer, *Theragra chalcogramma* (Alaska pollock), with 104 resources (Figs 1c–d and 3, Supplementary Table S3). Humans also exhibited the 3^rd^ and 5^th^ mean shortest path lengths of taxa in the intertidal and nearshore marine webs, respectively (Supplementary Table S4). Path length refers to the number of consumer or resource links that connect one species to another in a food web and is one simple indicator of how quickly effects can spread through a network^18^,^19^. The short path lengths reflect the fact that 94.4% and 95.9% of taxa in the intertidal and nearshore habitats, respectively, were within two feeding links of humans (Fig. 1e,f). *Homo sapiens* was also the 6^th^ and 15^th^ most omnivorous consumer taxon in the intertidal and nearshore marine food webs, respectively (Supplementary Table S5), feeding on everything from algae such as *Ulvaceae* (sea lettuce) to high trophic level species such as *E. jubatus* (Steller sea lion) and *Lamna ditropis* (salmon shark). The high levels of omnivory result in humans having mid-range trophic levels of 2.98 and 3.98, out of maxima of 3.98 and 4.94 in the intertidal and nearshore marine webs. In summary, Aleut foragers exceeded 97% to 100% of other taxa in several key topological roles (generality, omnivory, shortest path length) in the marine food webs of the Sanak intertidal and nearshore ecosystems.

### Human Feeding Behaviors and Potential Extinction Impacts

As a highly omnivorous “super-generalist” separated by only one or two trophic links from the vast majority of marine species, the Aleut population of *H. sapiens* which arrived, established, and persisted on the Sanak Archipelago over the last several thousand years was poised to have strong effects that could ripple throughout local ecosystems. Potential effects include depressing abundances of prey taxa, reorganizing community structure and function, and contributing to or causing short-term local extirpations or long-term extinctions. A few previous studies have suggested that the presence of humans in the greater Aleutian Island system was associated with shifting species body sizes, intermittent resource depression, local habitat reorganization, and short-term extirpations^12^,^13^,^20^ through both direct effects on prey and indirect effects on other species. However, there is little evidence that the presence of prehistoric humans was associated with long-term extinctions in the Aleutian Islands^21^. In our study system, there are no taxa in the Sanak zooarchaeological record that are not represented in the modern marine ecosystem^3^. It is possible that the now-extinct Steller’s sea cow, recorded in archaeological material from several more western Aleutian Islands, once inhabited the Sanak Archipelago, but there was no record of them in Sanak middens, and in any case their extinction did not occur in prehistoric times^13^,^14^.

In addition to the distinctive Aleut topological feeding roles characterized using the Sanak marine food web data, we considered two aspects of their feeding behavior that may have had strong impacts on extinctions. First, there is evidence that Aleut people shifted focus on particular resource taxa under different seasonal and environmental constraints, a behavior sometimes referred to as “prey switching.” For example, calmer weather allowed hunting of off-shore marine mammals such as phocids and otariids from kayaks or at distant haulouts^2^,^3^. Periods of stormy weather and rough seas would have rendered kayaking dangerous^22^,^23^, encouraging a switch to foraging for shellfish in the intertidal zone. These types of foraging shifts would have extended to other terrestrial and freshwater habitats not analyzed here due to less complete data. For example, during the summer salmon run, the Aleut of this region stopped foraging in the intertidal and nearshore marine ecosystems to focus on freshwater fishing of abundant salmon^3^,^22^,^23^. Such prey-switching behavior has been documented for other hunter-gatherer societies such as foraging peoples in the Arctic^24^. It is also similar to what has been observed for non-human generalist consumers in a variety of habitats. Prey-switching behavior, which has long been known to enhance ecologically stability^25^,^26^, can occur as a result of decreasing abundance and availability of a focal or preferred resource species, thus relaxing or removing predation pressure on highly impacted taxa and allowing them to recover. Prior simulations of food web dynamics have demonstrated a positive relationship between various implementations of this type of adaptive trophic behavior by consumers with multiple resources and the persistence of species in complex ecological networks^27^,^28^. Second, unlike other non-human consumers, Aleut used foraging technology that could increase their strength of feeding on some target resource species^15^. For example, the Aleut used technology such as kayaks, hooks, and spears^16^ that could make them strong and effective predators.

Using the “Allometric Trophic Network” (ATN) food web model of the nonlinear population biomass dynamics of many interacting species, we simulated the impacts of introducing an omnivorous, super-generalist, and potentially super-effective predator species on dynamically persistent food webs. The ATN model has often been used in idealized, exploratory ways similar to how it is used here (e.g.,^29^,^30^,^31^), but has also been successfully used to postdict seasonal biomass dynamics in a specific pelagic lake food web where rich temporally resolved data were available^32^. In our implementation, the introduced “human-like” species passively switches feeding among different prey as a result of changes in resource biomasses, in the same way as other consumers with multiple resources. To include the potential effectiveness of human foraging behavior as a result of use of technology, the ATN model was modified to allow the invading super-generalist (but not other consumers) to feed more strongly than expected by allometric scaling (i.e., body size) on a variable fraction of its potential prey.

**Figure 4:**
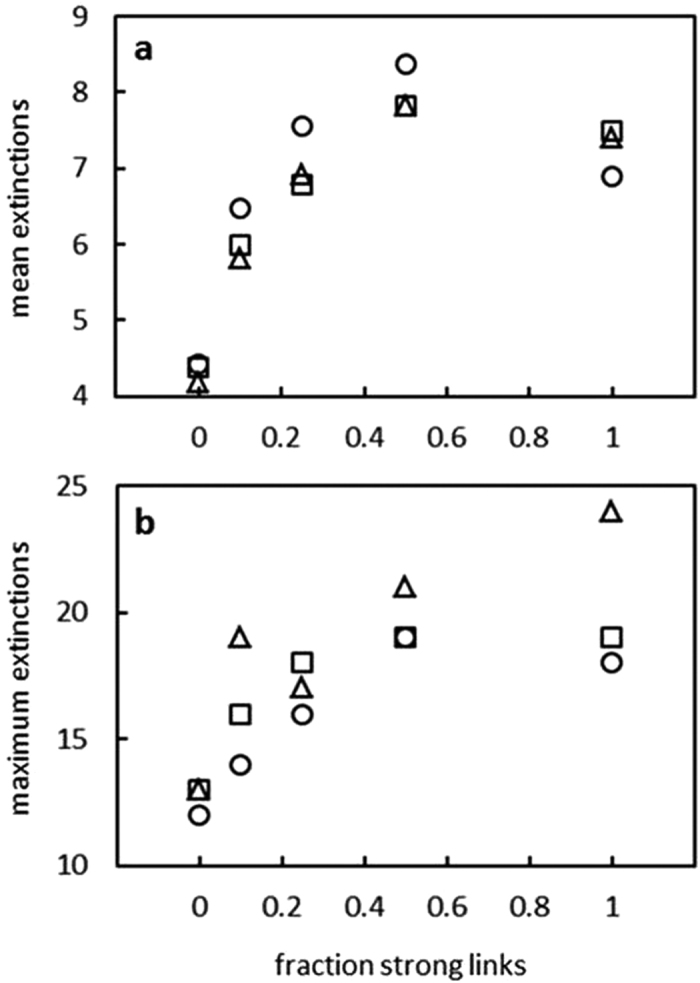
The impact of an invading generalist on persistence of taxa in food webs. The number of extinctions that occur when model food webs are invaded by a generalist that has a generality (fraction of taxa in the food web fed on) of 0.25 (circles), 0.50 (squares), 0.75 (triangles), as a function of the fraction of possible resource taxa the invading generalist feeds on strongly. (**a)** Mean number of extinctions. (**b)** Maximum number of extinctions. We do not include error bars as the intention of the simulations is to show the variability of the mean behavior of the model systems under different invasion conditions. Error bars that show the standard error of the mean become vanishingly small as the number of iterations of the model increases and so are of no interest. Error bars showing the standard deviation of the distribution just demonstrate variability across different individual runs of the model, and vary minimally in their magnitude across the different sets of model runs. Neither aspect of error is appropriate to, or informative of, the analysis presented here.

For ~100 dynamically persistent food webs examined with an average of 41 species, when the introduced super-generalist omnivore fed strongly on none of its potential prey, there were on average ~4 extinctions that followed. The average extinctions increased to ~6 with strong feeding on 10% of resources, and increased until they appear to saturate at ~8 given strong feeding on 50% of resources (Fig. 4a). Similar patterns emerged for maximum extinctions (Fig. 4b). These results suggest that while the introduction of a prey-switching and omnivorous super-generalist into an ecosystem is likely to lead to a small baseline number of extinctions, such impacts are exacerbated when strong feeding focuses on increasingly greater fractions of available resources. While other factors would likely have promoted or inhibited the long-term coexistence of species in the Sanak archipelago in the face of invasion and establishment of a human population that assumed distinctive trophic roles, such as climatic shifts or recruitment from regional species pools and associated metapopulation dynamics^33^,^34^, our simulations suggest that limited strong feeding behaviors by humans can enhance species persistence within local food webs. It will be interesting in future modeling work to understand the impact of more or less prey-switching in conjunction with different levels of strong feeding on species extinctions following the invasion of a human-like predator.

There is little doubt that prehistoric human hunter-gatherers^35^,^36^,^37^ and local indigenous commercial fisheries^38^ have affected marine ecosystems. Although humans have been a part of the North Pacific ecosystem for millennia, we have had little knowledge about how they fit into their ecosystems and what impacts they may have had on ecological function such as maintenance of biodiversity. By treating humans as part of, not external to, ecosystems, we can better understand the historical and current context of human roles in ecological systems. This study is a first attempt to use structural analysis and dynamical modeling of complex ecological networks to go beyond conventional notions of human impacts by investigating humans as one component of complex food webs in a region that supports some of the world’s last great fisheries. Our study suggests that even though human foragers in North Pacific marine ecosystems played special structural roles that could allow impacts of their feeding behavior to spread quickly throughout food webs potentially resulting in extinctions, limited technology-assisted strong foraging by a human population which prey-switched like other generalists would have promoted ecological integrity.

## Methods

### Study Site and Food Web Data

This project focuses on the Sanak Archipelago, 50 km south of the tip of the Alaska Peninsula. Sanak Island is about 120 km^2^ in area and has a 7,000-year archaeological record, which is 3000 years less than the record of the greater region, likely due to the remoteness of the archipelago. The coastline of the Sanak Archipelago contains a mix of semi-exposed rocky intertidal habitats, interspersed with protected sedimented and boulder-strewn shores. For the intertidal ecosystem, defined as benthic habitat up to the terrestrial vegetation exposed during low tide plus pelagic habitat above the benthos at high tide, we compiled species lists using a variety of field methods including randomly placed quadrats, sediment cores, and visual searches at locations spread across the entire archipelago over the span of three years. A full description of the methods used to catalog intertidal taxa is detailed in Wood *et al.* 2015^39^. For the nearshore marine ecosystem, defined as marine habitat within 80 km of the Sanak Archipelago, we inspected shallow benthic habitat (down to 30 feet below MLLW—mean lower low water, the average height of the lowest recorded tide each day) from the surface by snorkeling or shore- and boat-based observations, and augmented field observations with published occurrence records^40^,^41^,^42^,^43^,^44^,^45^,^46^,^47^,^48^,^49^,^50^,^51^,^52^,^53^,^54^,^55^,^56^,^57^,^58^,^59^. A majority of the taxa included in the nearshore web were reported by the Global Biodiversity Information Facility (GBIF;^39^) or Ocean Biographic Information System (OBIS;^39^) to exist within the bounding-box contained by 163.9°W/52.4°N and 160.2°W/55.0°N. Humans were included in both food webs. Several ubiquitous microscopic taxa were also assumed to be present in both habitats: Bacteria, Chrysophyta, Ciliophora, Cyanophycota, Dinophyceae, Euphausiidae, Foraminiferida, Phoronida, Prasinophyta, Prymnesiophyceae, Radiolaria and Sarcomastigophora. Avian taxa were included in either or both taxa lists based on observed or recorded extensive foraging in those habitats.

A total of 20,145 feeding links were observed in the field or compiled from descriptions in 1,532 peer-reviewed publications, dissertations, technical reports, or online databases, a more comprehensive version of the sources used in Wood *et al.* 2015^39^. Sources used a variety of methods to assess predation, including direct observation of feeding in the lab or field, gut or scat contents, and expert opinion. Human feeding links were identified via zooarchaeology of Sanak Island middens^3^, ethnographic data, and interviews with modern Unangan in the region. We strived for the highest possible taxonomic resolution in species identifications in the field and in the feeding data. When information about predators or prey was incomplete, or species could not be identified in the field, we lumped taxa into coarser-grained groups (e.g., genus or family) according to the taxonomy provided by the Integrated Taxonomic Information System^49^. The taxa and trophic link lists are available in Supplementary Data S1. The intertidal food web is a more resolved version of the Archipelago-wide food web presented by Wood *et al.* 2015^39^, which did not include humans. Trophic species versions of food webs (Supplementary Table. S1) were created by aggregating taxa in each web that have the same set of consumers and resources^60^.

These comprehensive, cumulative food webs represent the architecture of the feeding relationships among co-occurring taxa that lived or frequently fed in those habitats since human introduction. Although local abundances of taxa have shifted through time at scales that range from seasonal to hundreds of years, regional biodiversity, in terms of species presence/absence, has likely changed relatively little over the period of human habitation. For example, all marine species collected in Sanak Aleut middens are also present in the current intertidal or nearshore communities^3^. Even if a few species went locally extinct for periods of time, network structure analyses of food webs are robust to small changes in the inclusion or exclusion of particular nodes and their links^61^. Aspects of the diversity of the Sanak food webs were compared to those of 14 other coastal and marine webs^17^,^62^,^63^,^64^,^65^,^66^,^67^,^68^,^69^,^70^,^71^. Network structure analysis and visualization of the resulting intertidal and nearshore marine food webs was conducted using Network3D^72^,^73^.

### Food Web Dynamics Modeling

To simulate food web dynamics, we used the Allometric Trophic Network (ATN) model^30^,^31^,^32^. Using initial niche model structure^74^ with 50 species and a connectance of 0.15, we ran the ATN model with initial species biomasses chosen randomly between 0.1 and 1 and generated 105 dynamically persistent food webs (species biomass > 10^−10^ after 4000 time steps) with 33 to 47 taxa (mean = 41). Time steps are non-dimensional and scaled against the growth rate of the basal species, which have *r* = 1^76^. Generalist consumers feed on different resources in proportion to the resources’ relative biomasses^31^, which results in passive switching among possible prey. We invaded the webs with an omnivorous species (i.e., a predator that feeds on taxa randomly without regard to trophic level) at three high levels of generality—feeding on 25%, 50%, or 75% of the initial 50 taxa. The invading generalist fed strongly on different fractions of its possible prey—0, 0.1, 0.25, 0.5 and 1. The simulations were allowed to run for 3000 time steps, at which point we calculated the fraction of species that had gone extinct, defined as biomass dropping below the same extinction threshold used when creating the initial set of dynamically persistent webs^31^.

The ATN model was parameterized as in earlier work^31^,^75^ except that the predator-prey body-mass ratio was set to 100 and attack rate parameters were adjusted to model an invading species that feeds more strongly on some of its prey than expected by its body-mass-scaled rate parameters. In the ATN model, the functional response incorporates predators’ attack rates, which are critical for determining the strength of the feeding of predators on their prey. As in earlier work^31^,^75^, we used the functional response


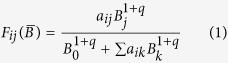


where *B*_0_ is the half-saturation density, *B*_*i*_ is the biomass of species *i*, and *q* = 0.2, which specifies a weak type III functional response. The relative attack rate of species *i* on species *j* is *a*_*ij*_, and the equations are scaled so that *a*_*ij*_ = 1/*n*_*i*_ where *n*_*i*_ is the number of prey of predator *i*. This sets generalist consumers to feed on different resources in proportion to the resources’ relative biomasses^31^,^75^, which results in passive switching among possible prey. Also, as the generality of a predator increases, its attack rate on any one prey item decreases. The species we introduced into each web was allowed to have a varying fraction of feeding links that were stronger than its other feeding links. The introduced species’ attack rate on those prey was *a*_*ij*_ = 3/*n*_*i*_, three times higher than the attack rate on other prey, and three times higher than the attack rate of other predators in the system.

## Additional Information

**How to cite this article**: Dunne, J. A. *et al.* The roles and impacts of human hunter-gatherers in North Pacific marine food webs. *Sci. Rep.* **6**, 21179; doi: 10.1038/srep21179 (2016).

## Supplementary Material

Supplementary Information

Supplementary Data S1
