# Supplementary material for: The roles and impacts of human hunter-gatherers in North Pacific marine food webs
Source: Sci Rep. 2016 Feb 17;6:21179. doi: 10.1038/srep21179 (PMC4756680; doi:10.1038/srep21179)
Supplement: Supplementary Information [file srep21179-s1.pdf]

Supplementary Information

The roles and impacts of human hunter-gatherers in North Pacific marine food webs

Jennifer A. Dunne, Herbert Maschner, Matthew W. Betts, Nancy Huntly, Roly Russell, Richard J. Williams, and Spencer A. Wood

This document contains the following supplementary information:

Supplementary Table S1 | Network structure properties of Sanak intertidal and nearshore food webs

Supplementary Table S2 | Previous food webs that include humans

Supplementary Table S3 | Top ten generalists of Sanak intertidal and nearshore food webs

Supplementary Table S4 | Ten taxa with shortest path lengths of Sanak intertidal and nearshore food webs

Supplementary Table S5 | Ten most omnivorous taxa of Sanak intertidal and nearshore food webs

Supplementary Figure S1 | Comparison of diversity and types of taxa in marine food webs

Supplementary References S1 | References associated with Supplementary Table S2 & Supplementary Figure S1

Supplementary Table S1

Network structure properties of Sanak intertidal and nearshore food webs

	Intertidal		Nearshore	
Property	Original	Trophic	Original	Trophic
S	235	232	513	511
L	1804	1799	6774	6771
L/S	7.7	7.8	13.2	13.3
C	0.033	0.033	0.026	0.026
Top	0.077	0.069	0.078	0.076
Int	0.749	0.759	0.813	0.816
Bas	0.174	0.172	0.109	0.108
Herb	0.174	0.177	0.133	0.133
Omn	0.634	0.642	0.735	0.738
Can	0.200	0.203	0.183	0.184
Loop	0.174	0.177	0.382	0.384
GenSD	1.27	1.26	1.24	1.24
VulSD	1.27	1.26	1.40	1.40
LinkSD	0.92	0.91	1.00	1.00
TroLev	2.44	2.44	2.83	2.83
MaxSim	0.41	0.40	0.37	0.37
Path	2.36	2.33	2.35	2.35
Clus	0.14	0.15	0.15	0.15

"Original" = original data, "Trophic" = trophic species versions of the webs. *S* = species richness, *L* = trophic links, *L/S* = link density, *C* = directed connectance (L/S^2). "Top", "Int", "Bas" are proportions of taxa that are top (lacking consumers), intermediate (have both consumers and resources), or basal (lacking resources). "Herb", "Omn", "Can", "Loop" are proportions of taxa that are herbivores, omnivores (i.e., feeding at multiple trophic levels), cannibals, and that occur in loops (e.g, A eats B eats C eats A). "GenSD", "VulSD", "LinkSD" are standard deviations of generality (number of resources), vulnerability (number of consumers), and total links (resources + consumers). "TroLev" is mean trophic level, calculated with the "short-weighted trophic level" algorithm. "MaxSim" refers to the mean maximum trophic similarity. "Path" refers to mean shortest path length, and "Clust" refers to clustering coefficient.

Supplementary Table S2

Previous food webs that include humans

Food Web	*S*	*L*	ECO	Ref
Cochin Backwater, India	9	18	1	1
Nearshore Marine, Aleutian Island, US	13	20	48	2
Lake Abaya, Ethiopia	13	24	77	3
Antarctic Seas	14	32	30	4
Salt Marsh, Rhode Island, US	15	25	8	5
Loch Leven 2, Scotland	15	22	118	6
Lake Rybinsk, Russia	16	32	71	7
Lake George, Uganda	16	27	78	8
Epipelagic Zone, Suruga Bay, Japan	16	37	86	9
Lake George, Uganda	16	19	120	10
Narragansett Bay, Rhode Island, US	20	34	7	11
Loch Leven 1, Scotland	22	32	68	12
Tidal Flat, California, US	25	44	6	13
Kapingamarangi Atoll, Polynesia	27	40	18	14
Ovre Heimdalsvatn Lake, Norway	27	48	126	15
Aspen Forest, Manitoba, Canada	34	58	26	16
Subtidal Rocky-Shore, Mediterranean Sea	37	151		17
Late Global Period, Adriatic Sea	39	302		18
Stream Bed, Aire, Nidd & Wharfe Rivers, Yorkshire, UK	60	185	210	19
Northeast US Shelf, US	81	1562		20

S is the number of taxa, *L* is the number of links. “Ref” gives the reference number (see Supplementary References S1) and “ECO” gives the number of the corresponding web in the ECOWeB database (21). Food webs are ordered by increasing *S*.

Supplementary Table S3

Top ten generalists of Sanak intertidal and nearshore food webs

	Sanak Intertidal Food Web				Sanak Nearshore Marine Food Web			
Rank	ID	Taxon	Name	#Res	ID	Taxon	Name	#Res
1	57	*Homo sapiens*	human	70	87	*Gadus macrocephalus*	Pacific cod	124
2	59	*Vulpes*	Arctic fox	50	179	*Homo sapiens*	humans	122
3	212	*Larus glaucescens*	glaucous-winged gull	47	89	*Theragra chalcogramma*	Alaska pollock	104
4	36	*Pycnopodia helianthoides*	sunflower sea star	39	177	*Hippoglossus stenolepis*	Pacific halibut	92
5	198	*Haliaeetus leucocephalus*	bald eagle	38	68	*Oncorhynchus tshawytscha*	Chinook salmon	85
6	161	*Cancer*	crab	37	192	*Eumetopias jubatus*	Steller sea lion	84
7	38	*Strongylocentrotus droebachiensis*	green sea urchin	34	142	*Myoxocephalus*	sculpin	71
8	200	*Falco peregrinus*	peregrine falcon	34	196	*Phoca vitulina*	harbor seal	68
9	58	*Enhydra lutris*	sea otter	32	66	*Oncorhynchus kisutch*	coho salmon	64
10	34	*Leptasterias*	six-rayed sea star	29	169	*Pleuronectes*	plaice/flounder	63

"ID" is the number of the node in Supplementary Dataset S1. "Taxon" is the scientific name of the taxon, "Name" is the common name of the taxon, "#Res" is the number of prey (resources) each taxon has.

Supplementary Table S4

Ten taxa with shortest path lengths of Sanak intertidal and nearshore food webs

	Sanak Intertidal Food Web				Sanak Nearshore Marine Food Web			
Rank	ID	Taxon	Name	Path	ID	Taxon	Name	Path
1	168	*Detritus*	detritus	1.63	353	*Gammaridea*	amphipod	1.65
2	150	*Gammaridea*	amphipod	1.73	382	*Detritus*	detritus	1.68
3	57	*Homo sapiens*	humans	1.76	89	*Theragra chalcogramma*	Alaska pollock	1.74
4	38	*Strongylocentrotus droebachiensis*	green sea urchin	1.81	87	*Gadus macrocephalus*	Pacific cod	1.74
5	161	*Cancer*	crab	1.82	179	*Homo sapiens*	human	1.80
6	64	*Bacillariophyta*	diatoms	1.84	375	*Cancer*	crab	1.82
7	212	*Larus glaucescens*	glaucus-winged gull	1.84	363	*Pandalidae*	shrimp	1.83
8	103	*Littorina*	periwinkle	1.87	224	*Clupea pallasii*	Pacific herring	1.85
9	59	*Vulpes*	Arctic fox	1.90	177	*Hippoglossus stenolepis*	Pacific halibut	1.85
10	127	*Mytilus*	mussel	1.91	217	*Hydrozoan*	hydrozoa	1.86

"ID" is the number of the node in Supplementary Dataset S1. "Taxon" is the scientific name of the taxon, "Name" is the common name of the taxon, "Path" is the mean shortest path length (i.e., the mean of the shortest chain of feeding interactions, regardless of direction, between each pair of species in the food web).

Supplementary Table S5

Ten most omnivorous taxa of Sanak intertidal and nearshore food webs

	Sanak Intertidal Food Web				Sanak Nearshore Marine Food Web			
Rank	ID	Taxon	Name	Omn	ID	Taxon	Name	Omn
1	181	*Branta canadensis*	Canada goose	1.15	152	*Eumicrotremus*	lumpsucker	1.17
2	160	*Telmessus cheiragonus*	helmet crab	0.88	359	*Thysanoessa longipes*	krill	1.04
3	159	*Scyra*	sharp-nosed crab	0.82	302	*Cephalaspidea*	sea slugs	1.03
4	154	*Hippolytidae*	shrimp	0.81	459	*Branta canadensis*	Canada goose	1.02
5	125	*Mopalia*	chiton	0.80	254	*Aphroditidae*	polychaete worms	1.00
6	57	*Homo sapiens*	humans	0.79	335	*Aetideidae*	copepod	0.92
7	67	*Foraminifera*	protists	0.78	264	*Thysanoessa raschii*	krill	0.91
8	38	*Strongylocentrotus droebachiensis*	green sea urchin	0.77	433	*Mopalia*	chiton	0.89
9	208	*Gallinago*	snipe	0.75	217	*Hydrozoa*	hydrozoan	0.87
10	153	*Euphausiidae*	krill	0.74	460	*Branta bernicla*	Brant goose	0.84

"ID" is the number of the node in Supplementary Dataset S1. "Taxon" is the scientific name of the taxon, "Name" is the common name of the taxon, "Omn" is the standard deviation of the trophic levels of all prey species of that taxon, a metric of omnivory.

Supplementary Figure S1

Comparison of diversity and types of taxa in marine food webs

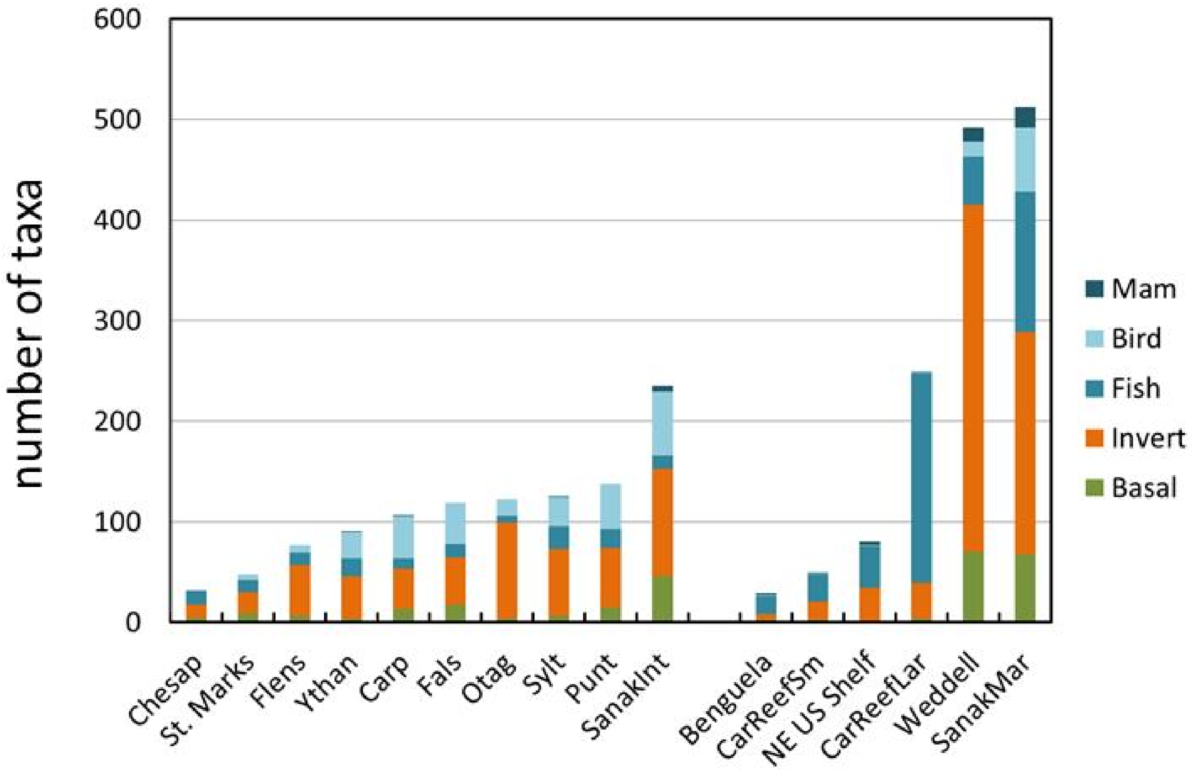

Comparison of diversity and types of taxa in marine food webs. First group shows data for ten coastal webs in order of increasing species richness: Chesap = Chesapeake Bay (22); St. Marks = St. Marks Estuary (23); Flens = Flensburg Fjord (24); Ythan = Ythan Estuary (25); Carp = Carpinteria Salt Marsh (26); Fals = Bahia Falsa (26); Otag = Otago Harbor (27); Sylt = Sylt Tidal Basin (28); Punt = Estero de Punta Banda (26); SanakInt = Sanak Intertidal. Second group shows six marine webs in order of increasing species richness: Benguela = Benguela Fishery (29); CarReefSm = Caribbean Reef, small version (30); NE US Shelf = Northeast U.S. Shelf (20); CarReefLar = Caribbean Reef, large version (30); Weddell = Weddell Sea (31); SanakMar = Sanak Nearshore. Vertebrate data shown in shades of blue, invertebrate in orange, basal in green. Data shown for original species versions of webs.

Supplementary References S1

References associated with Supplementary Table S2 & Supplementary Figure S1

1. Qazim, S. Z. Some problems related to the food chain in a tropical estuary in *Marine Food Chains* (ed. Steele, J. H.) 45-51 (Oliver and Boyd, 1970).
2. Simenstad, C. A., Estes, J. A. & Kenyon, K. W. Aleuts, sea otters, and alternate stable-state communities. *Science* **200**, 403-411 (1978).
3. Riedel, D. Der Margheritensee (Sudabessinien) - Zugleich ein Beitrag zur Kenntnis der Abessinischen Graben-Seen. *Arch Hydrobiol* **58**, 435-466 (1962).
4. Mackintosh, N. A. A survey of antarctic biology up to 1945 in *Biologie Antarctique* (ed. Carrick, R., Holdgate, M. & Prevost, J.) 3-38 (Hermann, 1964).
5. Nixon, S. W., Oviatt, C. A. Ecology of a New England salt marsh. *Ecol Mon* **43**, 463-498 (1973).
6. Blindloss, M. E., Holden, A. V., Bailey-Watts, A. E. & Smith, I. R. Phytoplankton production, chemical and physical conditions in Loch Leven in *Productivity Problems of Freshwaters* (ed. Kajak, Z., Hillbricht-Ilkowska, A.) 639-659 (Polish Scientific Publishers, 1972).
7. Sorokin, Y. I. Biological productivity of the Rybinsk reservoir in *Productivity Problems of Freshwaters* (ed. Kajak, Z., Hillbricht-Ilkowska, A.) 493-503 (Polish Scientific Publishers, 1972).
8. Burgis, M. J., Dunn, I. G., Ganf, G. G., McGowan, L. M. &Viner, A. B. Lake George, Uganda: Studies on a tropical freshwater ecosystem in *Productivity Problems of Freshwaters*, (ed. Kajak, Z., Hillbricht-Ilkowska, A.) 301-309 (Polish Scientific Publishers, 1972).
9. Hogetsu K Biological productivity of some coastal regions of Japan in *Marine Production Mechanisms,* International Biological Programme Series #20 (ed. Dunbar, M. J.) 71-87 (Cambridge University Press, 1979).
10. Moriarty, D. J. W., Darlington, J. P. E. C., Dunn, I. G., Moriarty, C. M. & Tevlin, M. P. Feeding and grazing in Lake George, Uganda. *Proc Roy Soc B* **184**, 299-319 (1973).
11. Kremer, J. N., Nixon, S. W. *A Coastal Marine Ecosystem: Simulation and Analysis*, Volume 24 of Ecological Studies (Springer-Verlag, 1978).
12. Morgan, N. C., McLusky, D. S. A summary of the Loch Leven IBP results in relation to lake management and future research. *Proc Roy Soc Edinburgh B* **74**, 407-416 (1972).
13. MacGinitie, G. E. Ecological aspects of a California marine estuary. *Am Midl Nat* **16**, 629-765 (1935).
14. Niering, W. A. Terrestrial ecology of Kapingamarangi Atoll, Caroline Islands. *Ecol Mon* **33**, 131-160 (1963).
15. Larson, P., Brittain, J. E., Lein, L., Lillehammer, A. & Tangen, K. The lake ecosystem of Ovre Heimdalsvatn. *Holar Ecol* **1**, 304-320 (1978).

16. Bird, R. D. Biotic communities of the Aspen Parkland of central Canada. *Ecology* **11**, 356-442 (1930).
17. Sala, E. The past and present topology and structure of Mediterranean subtidal rocky-shore food webs. *Ecosystems* **7**, 333-340 (2004).
18. Lotze, H. K., Coll, M. & Dunne, J. A. Historical changes in marine resources, food-web structure and ecosystem functioning in the Adriatic Sea, Mediterranean. *Ecosystems* **14**, 198-222 (2011).
19. Percival, E., Whitehead, H. A quantitative study of the fauna of some types of stream-bed. *J Ecology* **17**, 282-314 (1929).
20. Link, J. Does food web theory work for marine ecosystems? *Mar Ecol Prog Ser* **230**, 1-9 (2012).
21. Cohen, J. E. Ecologists' Co-Operative Web Bank Version 1.00. Machine-readable data base of food webs. (The Rockefeller University, 1989).
22. Baird, D. & Ulanowicz, R. E. The seasonal dynamics of the Chesapeake Bay ecosystem. Ecological Monographs 59, 329–364 (1989).
23. Christian, R. R. & Luczkovich, J. J. Organizing and understanding a winter's seagrass foodweb network through effective trophic levels. Ecological Modelling 117, 99–124 (1999).
24. Zander, C. D. et al. Food web including metazoan parasites for a brackish shallow water ecosystem in Germany and Denmark. Ecology 92, 2007 (2011).
25. Huxham, M., Beany, S. & Raffaelli, D. Do parasites reduce the chances of triangulation in a real food web? Oikos 76, 284-300 (1996).
26. Hechinger, R. F. et al. Food webs including parasites, biomass, body sizes, and life-stages for three California/Baja California estuaries. Ecology 92, 791 (2011).
27. Mouritsen, K .N., Poulin, R., McLaughlin, J. P. & Thieltges, D. W. Food web including metazoan parasites for an intertidal ecosystem in New Zealand. Ecology 92: 2006 (2011).
28. Thieltges, D. W., Reise, K., Mouritsen, K. N., McLaughlin, J. P. & Poulin, R. Food web including metazoan parasites for a tidal basin in Germany and Denmark. Ecology 92, 2005 (2011).
29. Yodzis, P. Local trophodynamics and the interaction of marine mammals and fisheries in the Benguela ecosystem. Journal of Animal Ecology 67, 635-658 (1998).
30. Opitz, S. Trophic Interactions in Caribbean Coral Reefs. (ICLARM Technical Report 43, 1996).
31. Jacob, U. et al. The role of body size in complex food webs: A cold case. Advances in Ecological Research 45, 181-223 (2011).
